# Relapse and Clinical Characteristics of Patients with Bipolar Disorders in Central Ethiopia: A Cross-Sectional Study

**DOI:** 10.1155/2020/8986014

**Published:** 2020-09-28

**Authors:** Habte Belete, Tilahun Ali, Getasew Legas

**Affiliations:** ^1^Psychiatry Department, College of Medicine and Health Sciences, Bahir Dar University, Bahir Dar, Ethiopia; ^2^Department of Psychiatry, School of Nursing and Midwifery, College of Health and Medical Sciences, Haramaya University, Ethiopia; ^3^Psychiatry Department, College of Medicine and Health Sciences, Debre Tabor University, Ethiopia

## Abstract

**Background:**

Bipolar disorder is a severe mental illness and has huge morbidity and mortality. Relapse is a challenging treatment failure in patients with mental illness, especially in patients with bipolar which causes high economic and social burdens. In the mental health delivery system, relapse is common and can be defined as becoming ill again after apparent recovery and a worsening condition of psychiatric patients. Due to psychiatric patients that may stop medication on their own in contrary to the advice of mental health professionals, relapse of mood episodes, delayed remission, and residual symptoms usually leads to hospitalization, increased suicide risk, and/or impede psychosocial recover. Therefore, understanding the nature of relapse in patients in low-income countries helps to prevent recurrence and related health care expenses.

**Objective:**

The objective of this study was to assess the prevalence and factors associated with relapse among patients with bipolar disorders in central Ethiopia.

**Method:**

Facility-based cross-sectional study was conducted from May to June 2015 at Amanuel Mental Specialized Hospital. Relapse was calculated among 400 samples of people with bipolar disorder, and systematic random sampling was used to select the study participants. Oslo's social support scale and ASSIST were used to identify factors with relapse, and a binary and multivariable logistic regression analysis model was performed to control the confounding factors. Odds ratios (OR) with the corresponding 95% confidence interval (95% CI) were determined to evaluate the strength of association.

**Results:**

The prevalence of relapse was 71% among patients with bipolar disorder. The longer morbidity (longer than 5 years) had a higher risk of relapse [adjusted odd ratio (AOR) = 3.91, 95% confidence interval (CI): 2.44 to 6.27], while good medication adherence found to be a protective factor for relapse [AOR = 0.39, 95% CI: 0.22 to 0.72].

**Conclusion:**

The prevalence of relapse was found pretty high among patients with bipolar disorders (71%). Working on treatment adherence and controlling the psychopathology is important to prevent relapse among bipolar patients.

## 1. Introduction

Bipolar disorder is a severe mental illness and has a huge morbidity and mortality in the community. Bipolar disorder is strongly associated with suicide ideation (59%), suicide attempts (56%), and up to 19% of patients with bipolar disorder die from suicide during their lifetime [1]. This burden is associated with relapse of the disorders and has a higher family burden to care patients with bipolar disorder than patients with other medical illnesses [2]. In the mental health delivery system, relapse means to become ill again after apparent recovery and a worsening condition of psychiatric patients [3]. Due to psychiatric patients that may stop medication on their own in contrary to the advice of mental health professionals, relapse of mood episodes, delayed remission, and residual symptoms usually leads to hospitalization, increased suicide risk, and/or impede psychosocial recover. Many clinical studies reported that stressful life events always precede the episodes of patients with bipolar disorders [4]. The family-focused psychoeducational treatment appears to be an efficacious adjunct to pharmacotherapy to prevent relapse [5, 6]. Less than optimum parenting and maltreatment histories have been associated with a worse course of bipolar disorders. Risk of relapse in bipolar individuals linked with the psychosocial context of illness, environmental, developmental, and cognitive-related factors [4]. Adjunctive psychological support with the usual psychiatric treatment has a significant reduction in relapse (of about 40%) compared to the standard treatment alone [7]. Relapse has a huge impact on the economic, interpersonal relationship and quality of life of patients as well as their family. Therefore, understanding the nature of relapse in patients in low-income countries helps to prevent recurrence and related health care expenses.

Magnitude of relapse varies in different studies. From a follow-up study, 48.5% of patients with bipolar disorder experienced recurrence. Among those patients with recurrence, 34.7% were manic relapses, and 13.8% were developing either depressive, hypomanic, or mixed episodes [8]. After one year of successful antidepressant response, the risk of depressive relapse in patients with bipolar disorder was 70% compared to those patients who discontinued antidepressants (36%) [9]. A follow-up study conducted indicated that the recovery rate of bipolar disorder was limited with syndromic recovery occurred in 48%, symptomatic recovery occurred in only 26%, and functional recovery in only 24% [10]. Relapse is higher in risky populations like pregnant women and sometimes it reaches up to 71% of mothers with bipolar disorder [11]. In this study, type of diagnosis, earlier onset, more recurrences/year, recent illness, had used antidepressants, and anticonvulsants versus lithium was found as the significant factors.

A community based study conducted in Ethiopia, lifetime prevalence of bipolar I disorder was estimated 0.6% for men and 0.3% for women [12]. From 312 patients with bipolar disorders, during follow-up, 65.9% of them experienced a relapse within 2.5 years (47.8% manic, 44.3% depressive and 7.7% mixed episodes, and 31.1% had persistent illness) [13]. In this study, female gender predicted depressive relapse, and male gender predicted manic relapse, while being on psychotropic medication that was associated with remission. Despite this higher relapse, there is no information in Ethiopian clinical settings on relapse among patients with bipolar disorders. Therefore, the aim of this study was to assess the magnitude of relapse and associated factors among patients with bipolar disorders at Amanuel Mental Specialized Hospital.

## 2. Methods and Materials

### 2.1. Study Design, Study Population, and Sampling

A cross-sectional study was conducted among bipolar patients who were attending at Amanuel Mental Specialized Hospital outpatient clinic. It has 15 outpatient departments in which 8 of them serve an average of 11,760 bipolar cases per year and other patients. Those patients who were clinically diagnosed (with Diagnostic and Statistical Manual of Mental Disorders IV-TR edition) for bipolar disorder (any type of bipolar disorder) were the source population. The study population was all patients who were attending outpatient clinics during the data collection time. Participants whose age 18 years and above were included. Patients who were incapable to communicate with data collectors were excluded. Sample was taken by considering 50% of the proportion of relapse in a single population proportion for unknown prevalence, with 5% error and 10% nonresponse rate. Among the 423 potential participants, 400 had completed the interview, but 10 denied participating, 8 failed to complete the interview, and 5 were excluded. The systematic random sampling technique was used to select study participants from 954 bipolar patients who visit the hospital per month averagely, and the sampling fraction was two. The data were collected by well-trained nurses by patient chart review and interviewing patients and their families using a questionnaire which is translated to a local language (Amharic, the national working language of Ethiopia), from May to June 2015.

### 2.2. Instrument

Data was collected by the structured interview questionnaire. We assessed relapse based on the DSM-IV-TR relapse definition criteria [14]. Bipolar patients and their primary caregivers were asked about the numbers of relapses. In addition to that, review was done to identify the recorded numbers of relapses. Study participant wealth was assessed by using principal component analysis in which eigenvalues greater than 1 were used as extractions, and the factors to extract were fixed at five (from lowest to highest). Current substance uses were assessed by adopted alcohol, smoking, and substance involvement screening test [15]; social support was assessed by the Oslo-3 Social Support Scale [16]; drug adherence was assessed by using the Medication Adherence Rating Scale (MARS) [17], and perceived stress was assessed by using of the Perceived Stress Scale 10 items tool [18] that focuses on patients who had experienced stressful life events. Psychopathological variables, such as current manic, depressive, and psychotic symptoms, were assessed by reviewing the patients' chart and self-report.

### 2.3. Data Collection Procedures

The questionnaire was pretested on a sample equal to 5% of the total sample size that were not part of the main study.

Face-to-face interviews and document review were used to collect the data for this study. Three BSc psychiatry nurses and three psychiatry postgraduate students attending their studies in the master's program in integrated community and clinical mental health (ICCM) were recruited as data collectors after the 3-day training was given, and the principal investigators supervised the data collection. Each data collector reviewed the card and recorded the card number of respondents who had completed the questionnaire and shared it to all the data collectors to avoid redundancy of the participants. The principal investigators and the supervisors monitored the data quality, checked completeness of the questionnaires, and feedback was given to data collectors on a daily base.

### 2.4. Data Analysis

Data were checked for completeness and entered to and clean up with Epi Data version 3.1. Then, after the double entry verification, the data were exported to SPSS version 20 for analyses. The dependent variable (relapse) and independent variables were entered into bivariate logistic regression in order to determine the statistical association between dependent and independent variables. All variables associated with the dependent variable with *P* − value less than 0.25 in the bivariate analyses of the binary logistic regression were entered into the multivariate models of the logistic regression by the enter method in order to identify the interaction between variables and to control for potential confounders. Multivariate logistic regression analysis was used to identify factors associated with relapse. Strength of association was presented by adjusted odd ratio (AOR) with 95% confidence interval (CI). *P* − value less than 0.05 in the multivariate analyses were used to select statistically significant variables.

## 3. Results

A total of 400 bipolar patients were successfully interviewed, and 228 (57%) of them were females. Majority of the participants, 287 (71.8%), were from the urban (Table 1).

### 3.1. Clinical Characteristics and Psychopathology of Patients

From the total, 146 (36.5%) of them had depressed feeling; 98 (24.5%) grandiosity and 119 (29.8%) had suspiciousness. From the total participants, 208 (52%) of them had high perceived stress, 133 (33.3%) of them had poor social supports, and 152 (38%) of them had poor family relationship due to their illness (Table 2).

### 3.2. Prevalence of Relapse

Among the total 400 patients, 284 (71%) of them had relapse. In terms of episode, depressive episode was the highest which was accounted for 192 (48%); manic episode was 98 (24.5%), and mixed episode was 104 (26%) of the total sample. Among the substance users, relapse was found common and in which 60/77 (77.9%) of tobacco user, 54/67 (80.6%) of khat chewers, and 85/112 (75.9%) of alcohol users had a relapse.

### 3.3. Contributing Factors

After multivariate analysis of relapse in relation to all independent variables, only poor drug adherence and longer duration of morbidity (longer than 5 years) were found to be statistically significant (Table 3).

## 4. Discussion

Prevention of relapse for mental disorders in low and middle-income countries recommended stepped care with a combined model for integration of drug and psychological treatments [6]. Prevalence of relapse was found higher than previous report, 65.9% in Ethiopia [12], similar with pregnancy mothers with bipolar disorder (71%) [10]. With methodological and setting differences, the current study clearly magnifies the current high magnitude of relapse in low-income settings.

Patients who had more than five years of morbidity had a higher chance of relapse [AOR = 3.91, 95% CI (2.44, 6.27)], which is contradicted from the previous community study. Even if bipolar disorders have a double risk of relapse than unipolar depression, in bipolar patients, the transition from states of remission to new episodes is linear and has a constant risk of recurrence over the lifespan [19]. This study supported by the previously community-based study which states that bipolar disorder may run a severe clinical course in developing country settings than in developed countries [12]. Even from the follow-up study, longer intake episodes predicted greater chronicity during the long-term follow-up period, and this leads to have a new onset of psychopathology in their course of the illness [20]. Patients who had good adherence to their medication had reported low chance of relapse [AOR = 0.39, 95% CI (0.22, 0.72)]. In this study, achieving a good treatment adherence found clinically important to prevent relapse and able to reduce by 61% of relapse among participants. Basically, improving medication adherence with delivery of psychoeducation and enhancement of treatment adherence is a necessary and promising management component to prevent relapse in patients with bipolar disorders [21]. Adherence to treatment for bipolar disorder should be managed by clinicians, and this may be enhanced by interventions that address issues of appropriately taking medications. Greatest desire outcomes working on adherence should be integrated into the medication management of bipolar illness, which helps to prevent relapse. Prevalence of relapse was found high and most importantly, patients who had longer than five years of morbidity found risk for relapse while those who had good adherence to their treatment was significantly protective. Finally, this study will initiate future studies in low-income countries concerning to mental health care and prevention of relapse.

### 4.1. The Strength and Limitation of the Study

Our study has several strengths. First, the study assesses the prevalence of relapse among bipolar patients, which was not previously studied. Second, we used validated and standardized tool for the assessment of independent variables. Since the cross-sectional study design could not establish clear risks of relapse in bipolar patients and does not test time to event and the exact time of relapse was not assessed after onset of the illness.

## 5. Conclusion

The prevalence of relapse was found pretty high among patients with bipolar disorders (71%). Working on treatment adherence and controlling the psychopathology are important to prevent relapse among bipolar patients.

## Figures and Tables

**Table 1:** Sociodemographic factors of the participants at the Amanuel Mental Specialized Hospital (*n* = 400), 2015.

Characteristics	Frequency	Percent (%)
Sex		
Male	172	43
Female	228	57
Living area		
Urban	287	71.7
Rural	113	28.3
Age		
18-24	74	18.5
25-34	154	38.5
35-44	104	26
45 -54	42	10.5
55 and above	26	6.5
Education		
Unable to read and write	65	16.3
Educated	335	83.7
Religion		
Orthodox	236	59
Muslim	99	24.7
Protestant	59	14.8
Catholic	6	1.5
Marital status		
Unmarried	225	56.2
Married	175	43.8
Ethnicity		
Amhara	139	34.7
Oromo	132	33
Gurage	79	19.8
Tigray	22	5.5
Others^∗^	28	7
Job		
Employed	293	73.2
Unemployed	107	26.8
Living condition		
With family	349	87.2
Alone	51	12.8
Wealth index		
Lowest	80	20.0
Second	85	21.3
Middle	75	18.7
Fourth	86	21.5
Highest	74	18.5

^∗^Others (Wolaita, Sidama, Gamo).

**Table 2:** Clinical characteristics and current psychopathology of patients with bipolar disorder (*n* = 400) at the Amanuel Mental Specialized Hospital, 2015.

Variable	Frequency	Percentage (%)
Age at the onset of 1^st^ episode		
Less than 18 years	67	16.8
18 to 24 years	155	38.7
Older than 24 years	178	44.5
Mood stabilizer		
Yes	304	76
No	96	24
Antidepressant		
Yes	56	14
No	344	86
Antipsychotic		
Yes	259	64.7
No	141	35.3
Social support (Oslo-3)		
Poor	133	33.2
Moderate	182	45.5
Strong	85	21.3
Duration of morbidity		
Up to five years	189	47.3
Longer than five years	211	52.7
Perceived stress (PSS)		
Low	109	27.2
Average	83	20.8
High	208	52
Medication adherence (MARS)		
Low	109	27.3
Average	166	41.5
High	125	31.2
Alcohol consumption		
Yes	112	28
No	288	72
Tobacco smoking		
Yes	77	19.3
No	323	80.7
Khat chewing		
Yes	67	16.8
No	333	83.2
Somatic symptoms		
Yes	37	9.3
No	363	90.7
Anxiety symptoms		
Yes	132	33
No	268	67
Perceptual disturbance		
Yes	57	14.3
No	343	85.7
Guilty feeling		
Yes	142	35.5
No	258	64.5
Mannerism		
Yes	60	15
No	340	85
Grandiosity		
Yes	98	24.5
No	302	75.5
Depressive symptoms		
Yes	146	36.5
No	254	63.5
Hostility		
Yes	100	25
No	300	75
Suspiciousness		
Yes	119	29.8
No	281	70.2
Hallucination		
Yes	63	15.8
No	337	84.2
Psychomotor disturbance		
Yes	43	10.8
No	357	89.2

MARS: Medication Adherence Rating Scale; Oslo-3: Oslo Social Support Scale; PSS: Perceived Stress Scale.

**Table 3:** Factors associated with relapse among patients with bipolar disorder at the Amanuel Mental Specialized Hospital psychiatric outpatient departments (*n* = 400), 2015.

Explanatory variables	Relapse		Adjusted odd ratio (95% CI)	*P* value
	Yes	No		
Living circumstance				
With family	247	102	1.00	0.804
Alone	37	14	0.91 (0.44, 1.9)	
Sex				
Male	124	48	1.00	0.710
Female	160	68	0.91 (0.57, 1.47)	
Job				
Employed	207	86	1.00	0.642
Unemployed	77	30	1.14 (0.66, 1.96)	
Age				
18-24	45	29	1.00	
25-34	102	52	1.13 (0.59, 2.14)	0.717
35-44	78	23	1.62 (0.76, 3.43)	0.209
45 -54	34	8	2.27 (0.83, 6.2)	0.111
55 and above	22	7	2.52 (0.72, 8.85)	0.149
Marital status				
Married	119	56	1.00	0.197
Unmarried	165	60	1.36 (0.85, 2.16)	
Poor family relation				
Yes	166	82	1.45 (0.89, 2.37)	0.135
No	118	34	100	
Medication adherence				
Poor	88	21	1.00	
Moderate	107	59	0.56 (0.29, 1.10)	0.076
Good	89	36	0.39 (0.22, 0.72)^∗^	0.002
Duration of illness				
≤5 years	108	81	1.00	
>5 years	176	35	3.91 (2.44, 6.27)^∗^	0.0001

^∗^Statistically significant.

## Data Availability

The data used to support the findings of this study are included within the article.

## Abbreviations

AOR: Adjusted odd ratio
CI: Confidence interval
MARS: Medication Adherence Rating Scale
Oslo-3: Oslo Social Support Scale
PSS: Perceived Stress Scale.

## References

[1] de Abreu L. N., Lafer B., Baca-Garcia E., Oquendo M. A. (2009). Suicidal ideation and suicide attempts in bipolar disorder type I: an update for the clinician. *Revista brasileira de psiquiatria*.

[2] Zergew A., Hailemariam D., Alem A., Kebede D. (2008). A longitudinal comparative analysis of economic and family caregiver burden due to bipolar disorder. *African Journal of Psychiatry*.

[3] Mwaba K., Molamu R. B. (1998). Perceived causes of relapse among a sample of recovering psychiatric patients at a Mafikeng hospital. *Curationis*.

[4] Alloy L. B., Abramson L. Y., Urosevic S., Walshaw P. D., Nusslock R., Neeren A. M. (2005). The psychosocial context of bipolar disorder: environmental, cognitive, and developmental risk factors. *Clinical psychology review*.

[5] Miklowitz D. J., Simoneau T. L., George E. L. (2000). Family-focused treatment of bipolar disorder: 1-year effects of a psychoeducational program in conjunction with pharmacotherapy. *Biological Psychiatry*.

[6] Patel V., Araya R., Chatterjee S. (2007). Treatment and prevention of mental disorders in low-income and middle-income countries. *The Lancet*.

[7] Scott J., Colom F., Vieta E. (2007). A meta-analysis of relapse rates with adjunctive psychological therapies compared to usual psychiatric treatment for bipolar disorders. *International Journal of Neuropsychopharmacology*.

[8] Perlis R. H., Ostacher M. J., Patel J. K. (2006). Predictors of recurrence in bipolar disorder: primary outcomes from the systematic treatment enhancement program for bipolar disorder (STEP-BD). *American Journal of Psychiatry*.

[9] Altshuler L., Suppes T., Black D. (2003). Impact of antidepressant discontinuation after acute bipolar depression remission on rates of depressive relapse at 1-year follow-up. *American Journal of Psychiatry*.

[10] Keck P. E., McElroy S. L., Strakowski S. M. (1998). 12-month outcome of patients with bipolar disorder following hospitalization for a manic or mixed episode. *American Journal of Psychiatry*.

[11] Viguera A. C., Whitfield T., Baldessarini R. J. (2007). Risk of recurrence in women with bipolar disorder during pregnancy: prospective study of mood stabilizer discontinuation. *American Journal of Psychiatry*.

[12] Negash A., Alem A., Kebede D., Deyessa N., Shibre T., Kullgren G. (2005). Prevalence and clinical characteristics of bipolar I disorder in Butajira, Ethiopia: a community-based study. *Journal of affective disorders.*.

[13] Fekadu A., Kebede D., Alem A. (2006). Clinical outcome in bipolar disorder in a community-based follow-up study in Butajira, Ethiopia. *Ethiopia. Acta Psychiatrica Scandinavica*.

[14] Stahl S. M. (2000). *Essential Psychopharmacology: Neuroscientific Basis and Practical Applications*.

[15] Humeniuk R., Ali R., Babor T. F. (2008). Validation of the alcohol, smoking and substance involvement screening test (ASSIST). *Addiction*.

[16] Bøen H. (2012). Characteristics of senior centre users–and the impact of a group programme on social support and late-life depression. *Norsk epidemiologi*.

[17] Fialko L., Garety P. A., Kuipers E. (2008). A large-scale validation study of the medication adherence rating scale (MARS). *Schizophrenia research*.

[18] Cohen S., Kamarck T., Mermelstein R. (1983). A global measure of perceived stress. *Journal of health and social behavior*.

[19] Angst J., Gamma A., Sellaro R., Lavori P. W., Zhang H. (2003). Recurrence of bipolar disorders and major depression. *European archives of psychiatry and clinical neuroscience*.

[20] Judd L. L., Akiskal H. S., Schettler P. J. (2002). The long-term natural history of the weekly symptomatic status of bipolar I disorder. *Archives of general psychiatry.*.

[21] Berk L., Hallam K. T., Colom F. (2010). Enhancing medication adherence in patients with bipolar disorder. *Human Psychopharmacology: Clinical and Experimental*.

